# Hydrothermal Synthesis of Ni_3_TeO_6_ and Cu_3_TeO_6_ Nanostructures for Magnetic and Photoconductivity Applications

**DOI:** 10.1021/acsanm.3c00630

**Published:** 2023-03-09

**Authors:** Javier Fernández-Catalá, Harishchandra Singh, Shubo Wang, Hannu Huhtinen, Petriina Paturi, Yang Bai, Wei Cao

**Affiliations:** † Nano and Molecular Systems Research Unit, University of Oulu, Oulu FIN-90014, Finland; ‡ Materials Institute and Inorganic Chemistry Department, University of Alicante, Ap. 99, E-03080 Alicante, Spain; § Wihuri Physical Laboratory, Department of Physics and Astronomy University of Turku, Turku FIN-20014, Finland; ∥ Microelectronics Research Unit, Faculty of Information Technology and Electrical Engineering, University of Oulu, FI-90570 Oulu, Finland

**Keywords:** transition metal tellurates, hydrothermal synthesis, optical properties, photoconductivity, magnetism

## Abstract

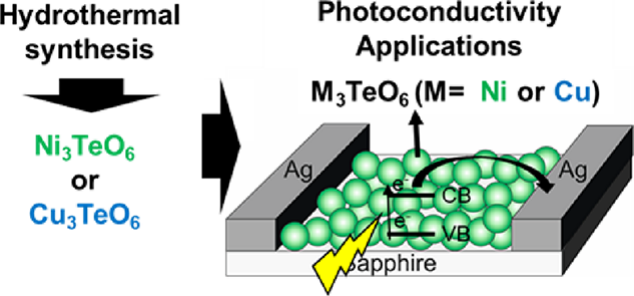

Despite great attention toward transition metal tellurates especially M_3_TeO_6_ (M = transition metal) in magnetoelectric applications, control on single phasic morphology-oriented growth of these tellurates at the nanoscale is still missing. Herein, a hydrothermal synthesis is performed to synthesize single-phased nanocrystals of two metal tellurates, i.e., Ni_3_TeO_6_ (NTO with average particle size ∼37 nm) and Cu_3_TeO_6_ (CTO ∼ 140 nm), using NaOH as an additive. This method favors the synthesis of pure NTO and CTO nanoparticles without the incorporation of Na at pH = 7 in MTO crystal structures such as Na_2_M_2_TeO_6_, as it happens in conventional synthesis approaches such as solid-state reaction and/or coprecipitation. Systematic characterization techniques utilizing in-house and synchrotron-based characterization methods for the morphological, structural, electronic, magnetic, and photoconductivity properties of nanomaterials showed the absence of Na in individual particulate single-phase MTO nanocrystals. Prepared MTO nanocrystals also exhibit slightly higher antiferromagnetic interactions (e.g., *T*_N_-NTO = 57 K and *T*_N_-CTO = 68 K) compared to previously reported MTO single crystals. Interestingly, NTO and CTO show not only a semiconducting nature but also photoconductivity. The proposed design scheme opens the door to any metal tellurates for controllable synthesis toward different applications. Moreover, the photoconductivity results of MTO nanomaterials prepared serve as a preliminary proof of concept for potential application as photodetectors.

## Introduction

Transition metal tellurates in particular M_3_TeO_6_ (MTO; M = Ni, Co, Mn, Cu) exhibit a broad variety of technologically important properties. Most of reported results focus on antiferromagnetic ordering at low temperatures combined with magnetoelectric properties.^1−3^ They are primarily classified as type II multiferroics and have recently gained great importance.^2,4−6^ For instance, the longest known tellurate of this family is Ni_3_TeO_6_ (NTO), which was structurally investigated in the year 1967.^7^ NTO crystallizes in corundum (Al_2_O_3_ type) structures with a polar R3 space group geometry. Ni cations in NTO reside in three different crystallographic sites and form face-shared, edge-shared, and corner-shared distorted NiO_6_ octahedra. TeO_6_ also forms a distorted octahedron. While the magnetic property of NTO at room temperature does not seem interesting, the low-temperature characteristic of NTO is intriguing and has drawn tremendous interest due to its unusual spin-flop transition, a complex magnetic field temperature phase diagram, and the largest magnetoelectric coupling.^8^ Recently, spin-driven pyroelectricity (colossal magnetoelectric effect) below the antiferromagnetic (AFM) ordering temperature of *T*_N_ = 52 K was reported for NTO, together with an exceptional possibility of a magnetoelectric switching without hysteresis.^1,2,4^ A few more reports summarize that already small changes in electric and magnetic fields are sufficient for a spin-flop transition in NTO particles.^8−10^ Furthermore, Cu_3_TeO_6_ (CTO) crystallizes in the cubic system of space group Ia-3 and exhibits an interesting structural feature. Each TeO_6_ octahedron connects to 12 distorted CuO_6_ octahedra, forming a unique three-dimensional framework. Three notable spin lattices, namely, equilateral triangle, isosceles triangle, and planar hexagon, are built by CuO_6_ octahedra via corner-sharing or edge-sharing. Concerning the low-temperature AFM property, CTO adopts a bixbyite-type crystal structure and orders in a “three-dimensional spin web” with hexagonal arrangements of the magnetic moments below *T*_N_ = 63 K.^3,11,12^ Besides these reports, which mainly focus on magnetoelectric application, an additional report also explores the photocatalytic activity^13^ and electrochemical properties^14^ of NTO and their application as sensor of CTO,^15^ indicating a possible bigger picture of MTO with nanoscale features.

While the role of transition metals in several transition metal-based complexes including MTO has been already been studied,^16^ the role of tellurium (Te) in such compounds is still a paradox. Te, a chalcogenide high-Z element, has already provided great development potential in the fields of high-performance photodetectors, field effect transistors, and thermoelectric devices in recent years.^17,18^ However, the similar device potential for transition metal-based tellurium oxide is still critical.^15^ Te-based compounds, in particular a combination of transition metals, Te, and oxygen (referred to here as tellurate, i.e., MTO), have recently been reported to be a growing material class owing to the higher electronic conductivity of Te. It has recently been seen to concur with the electronic conductivity issue in transition metal-based complexes when combined with Te toward wider energy applications;^19,20^ however, it is rarely engineered and implemented for morphology-dependent low-dimensional nanostructures for photonics, optoelectronics, and/or catalytic applications. Most of the research on such combination, for example MTO, has been dedicated to the condensed matter physics side such as magnetic properties due to the presence of magnetic elements (M = Ni, Co, Mn, Cu in MTO, all having responsible free electrons in their d orbital). However, the intrinsic features of Te, mainly higher electronic conductivity and its chalcogen nature, have been overlooked. Inclusion of these characteristics of M and Te in MTO might result in additional applications aforesaid above and is explored in this work.

Previously, MTO such as NTO and CTO has been grown mostly using conventional synthesis approaches such as solid-state reaction and/or coprecipitation methods.^2,21,22^ These processes show challenges in getting single-phase MTO materials probably because of the structural diversity of Te-based compounds and a wide range of possible Te–O bond lengths and in the size of the particles due to the high temperatures used.^23,24^ Aforesaid potential applications and reported complicated synthesis demand a cost-effective and facile approach for MTO with improved performances and are the focus of the present work. Alternative synthesis of MTO materials can be found in wet chemical methodologies (sol–gel or co-precipitation) to enable the materials’ applications in photocatalysis,^13^ electrocatalysis,^25^ and sensors.^15^ Indeed, the hydrothermal approach benefits wet synthetic routes and is widely used in ceramics synthesis.^26,27^ This methodology presents several advantages, i.e., excellent control of the composition and the size distribution at the nanoscale, compared to solid-state synthetic methodologies.^28^ This composition control is an important parameter in the synthetic procedures of novel ceramic materials,^29−31^ such as MTOs, since the incorporation of additives, such as NaOH, to control the pH and the crystal growth mechanism can produce different phases in the final product, such as Na_2_M_2_TeO_6_ (M = Ni or Cu).^32,33^ Consequently, the design of novel synthetic strategies of MTOs, with optical, photoconductivity, and magnetic properties using additives (NaOH) and novel procedures (hydrothermal synthesis), is crucial to opening the door to this family of metal tellurates for different applications. Nevertheless, it is admitted that the insufficient reserve of Te on the earth may be a drawback for the application of the materials studied in this work.^34,35^ It is thus important to consider the abundancy of the precursors in future works.

With this in mind, in this work two metal tellurates based on Ni (NTO) and Cu (CTO) have been synthesized using the hydrothermal method by adding NaOH to control the pH and increase the growth speed of MTO crystals. The materials synthesized have been systematically characterized by utilizing in-house and synchrotron-based characterization methods for their morphological, structural, electronic, magnetic, and photocatalytic properties. The NTO and CTO synthesized using hydrothermal methodology presented optical, photoconductivity, and magnetic properties. The measured current–voltage response in the dark and under illumination with different wavelengths serves as a proof of concept for potential applications in photodetection. The magnetic transition at low temperatures demonstrates a potential application as a thermal-magnetic sensor. Moreover, the synthetic methodology used for MTO may also be transferred to their chalcogen analogues (Se, P, and S), which broadens the applicability.

## Experimental Section

### Materials

Copper nitrate (Cu(NO_3_)_2_·3H_2_O, 99–104%, Sigma-Aldrich), nickel nitrate (Ni(NO_3_)_2_·6H_2_O, 98%, Alfa Aesar), telluric acid (H_6_O_6_Te, 98%, Sigma-Aldrich), sodium hydroxide (NaOH, 97%, Sigma-Aldrich), absolute ethanol (EtOH, 99.5%, ETAX), and deionized water were used in the present work. All reactants were used as received, without any further purification.

### Materials Preparation

**Scheme 1:**
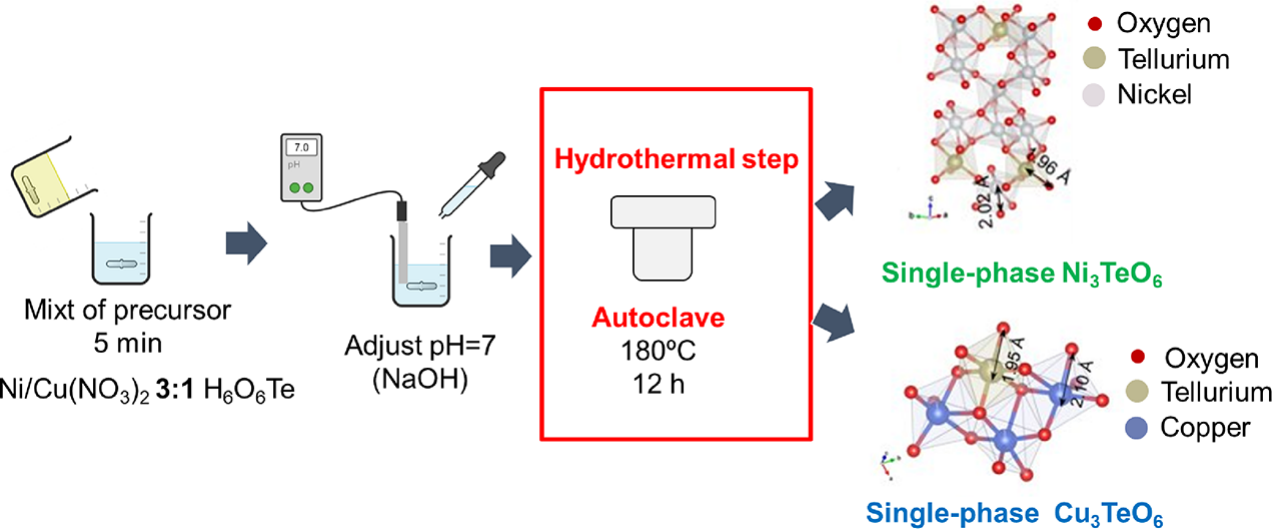
Hydrothermal Synthetic Procedure Followed for the Preparation of Single-Phase Ni_3_TeO_6_ and Cu_3_TeO_6_ Nanostructures

Ni_3_TeO_6_ and Cu_3_TeO_6_ materials were prepared by hydrothermal synthesis. For the synthesis of Ni_3_TeO_6_ and Cu_3_TeO_6_, using hydrothermal synthesis and NaOH as an additive (see Scheme 1), appropriate amounts of reagents, Ni(NO_3_)_2_·6H_2_O or Cu(NO_3_)_2_·3H_2_O, and H_6_O_6_Te, were mixed in a stoichiometric ratio (3:1, respectively) in deionized (DI) water (60 mL). This solution was stirred vigorously for 10 min. Then, NaOH (2 M) solution was used to adjust the pH to 7 in vigorous stirring. The mixture adjusted to pH = 7 was stirred for 5 min. This solution was transferred to an autoclave (100 mL) and heated up to 180 °C for 12 h. The mixtures were centrifuged (5000 rpm) to collect the solid materials, and they were washed three times with DI water and three times more with EtOH in order to clean the samples from some impurities obtained during the synthetic process. The solid obtained was calcined at 600 °C for 2 h. The as-obtained Ni_3_TeO_6_ and Cu_3_TeO_6_ powders are named as NTO_H, and CTO_H, respectively.

For comparison, Ni_3_TeO_6_ and Cu_3_TeO_6_ materials were prepared using the co-precipitation method. For the latter approach, appropriate amounts of reagents Ni(NO_3_)_2_·6H_2_O or Cu(NO_3_)_2_·3H_2_O and H_6_O_6_Te were mixed in a stoichiometric ratio in DI water (60 mL). This solution was stirred vigorously for 10 min. Then, NaOH (2 M) solution was used to adjust the pH to 7. The mixture was stirred for 8 h at 80 °C on a hot plate. The mixtures were centrifuged (5000 rpm) to collect the solid materials, and they were washed three times with DI water and three times more with EtOH in order to clean the samples of some impurities obtained during the synthetic process. The solid obtained was calcined at 600 °C for 2 h. Ni_3_TeO_6_ and Cu_3_TeO_6_ obtained by co-precipitation synthesis are named as NTO_CP and CTO_CP, respectively.

### Materials Characterization

Powder XRD patterns at room temperature were performed with Rigaku SmartLab 9 kW equipped with a five-axis θ–θ goniometer and a 1D solid-state detector and scintillator using Co Kα (λ = 1.79 Å, 40 kV, 135 mA) radiation. To obtain microstructures of the bulk, synchrotron X-ray diffraction (SXRD) measurements were carried out at the Brockhouse High Energy Wiggler Beamline (Canadian Light Source (CLS), Canada).^36^ The data acquisition was performed in transmission mode, so a 2D Perkin Elmer detector, 200 × 200 μm^2^ in pixel size and 40 × 40 cm^2^ in area, was placed downstream of the powders in a Kapton capillary. A monochromatic focused beam of 30 keV was used to obtain the patterns. Different from in-house powder XRD, SXRD owns larger penetration depths, thus probing the microstructures of the whole particles. The calibrated X-ray wavelength and sample-to-detector distance from a Ni calibrant were λ = 0.4087 Å and 478.8 mm, respectively. A total of 64 snapshots were acquired, and an exposure time of 0.2 s was used to ensure a good data statistic. The obtained raw 2D diffraction patterns were integrated in the radial direction by GSAS-II software;^37^ the resulting 1D SXRD profiles were analyzed using Rietveld refinement analysis.^38^ Transmission electron microscopy (TEM) coupled with electron energy loss spectroscopy (EELS) and energy-dispersive spectroscopy (EDS) mapping was carried out using JEOL JEM-2200FS EFTEM/STEM. Field emission scanning electron microscope (FESEM) images were taken using Zeiss Ultra Plus FESEM. The specific surface areas, average pore sizes, and volumes of the synthesized materials were measured with N_2_ adsorption at −195 °C using a micrometrics ASAP 2020 surface analyzer. Before the analyses, the samples were evacuated for 4 h at 250 °C. Optical spectroscopies were obtained using a Shimadzu UV-2600 spectrophotometer. XPS measurements were performed with Al Kα using Thermo Fisher Scientific ESCALAB 250Xi XPS System. Energy calibration of the XPS was performed by using the C 1s peak at 284.8 eV.

### Magnetic Property Measurements

The temperature dependence of the zero-field-cooled (ZFC) and field-cooled (FC) magnetization was measured for the powder samples between the temperatures of 5 and 300 K with a Quantum Design MPMS XL SQUID magnetometer with the external magnetic field of *B* = 10 mT, 100 mT, and 5 T. The magnetic hysteresis curves were recorded in magnetic fields up to 5 T at temperatures of 5 and 300 K.

### Device Fabrication and Photoconductivity Measurements

To assess the feasibility of being used in photodetectors, the synthesized NTO and CTO were made into a conventional, transverse sensor configuration. The NTO and CTO nano-powders were deposited on sapphire substrates. The methodology used was drop coating using dispersion of NTO or CTO (50 mg) in EtOH (5 mL). The drops deposited on sapphire substrates were dried at 80 °C. Ag electrodes were then coated on the sample surfaces to form in-plane electrode configurations with approximately 700 μm gaps. The size of the lab device was 1 × 1 cm. Current–voltage (*I*–*V*) curves were obtained using the 2450 SourceMeter (Keithley, USA) in the dark and under different incident photon energies, i.e., 1.88, 2.25, and 3.06 eV. Monochromatic lasers (OBIS LX/LS series, Coherent, USA) with wavelengths of 660, 552, and 405 nm, respectively, were used as the light sources. The power density of the incident lights was about 4 W/cm^2^. Sheet resistance was calculated from the *I*–*V* curves.

## Results and Discussion

### Crystal, Morphological, and Electronic Structures

**Figure 1:**
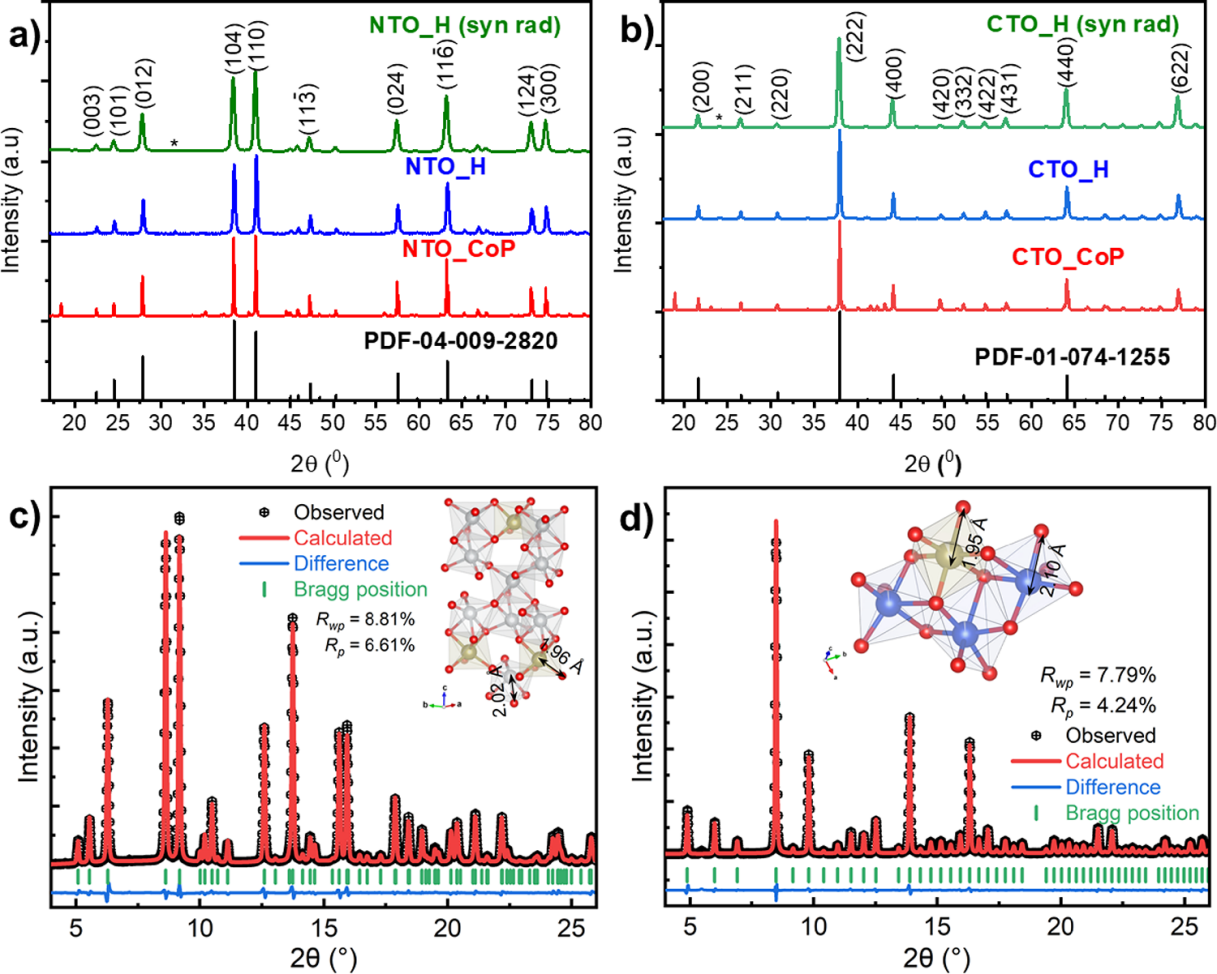
PXRD patterns for synthesized NTO and CTO samples: (a) NTO samples and (b) CTO samples. Rietveld refinement results for SXRD profiles and polyhedral view from fitted data of (c) NTO_H and (d) CTO_H.

To study the crystal structure of the metal tellurates (NTO and CTO) synthesized by hydrothermal synthesis, the final powders of the synthesis were characterized by lab-source XRD analysis (Figure 1). Sample NTO_H shows characteristic diffraction peaks at 2θ = 22.50, 24.55, 27.84, 38.487, 41.04, 47.31, 57.52, 63.27, 73.07, and 74.78°, which correspond to the characteristic peaks of (003), (101), (012), (104), (110), (113̅), (024), (116̅), (124), and (300) lattice planes of Ni_3_TeO_6_ (PDF 04-009-2820) (see Figure 1a). This comparative XRD result indicates that the phased NTO is synthesized without incorporation of Na in the crystalline structure of metal tellurate. However, the NTO_H diffraction patterns (Figure 1a) present a small broad peak at 31.08°, indicating possible impurities of another metal tellurate, NiTe_2_O_5_ (PDF 00-027-1306). With the aim of deeply studying the crystalline structure of NTO, the sample was analyzed by synchrotron radiation XRD and converted to the same 2θ angle scale as lab-source X-ray (Figure 1a).^24,39^ Under synchrotron radiation, XRD NTO_H shows mainly characteristic diffraction peaks of Ni_3_TeO_6_ (PDF 04-009-2820), indicating that this is the main phase. However, a small peak at 31.62° is observed indicating the presence of a NiTe_2_O_5_ impurity. The appearance of NiTe_2_O_5_ in low amount can be ascribed to the calcination step, taking into account that this phase can be generated by the Ni_3_TeO_6_ decomposition at high temperature.^25,40^ This fact highlights the relevance of using synchrotron radiation XRD to characterize the crystalline structure of inorganic materials.^41^ The CTO_H samples (Figure 1b) show characteristic diffraction peaks at 2θ = 20.93, 25.90, 30.10, 37, 25, 43.42, 54.07, 56.52, 63.48, 70.05, and 76.377°, which correspond to the characteristic peaks of (200), (211), (220), (222), (400), (422), (431), (440), (600), and (622) lattice planes of Cu_3_TeO_6_ (PDF 01-074-1255), respectively, related to the Cu_3_TeO_6_ crystalline single phase. The CTO_H sample was analyzed by synchrotron radiation XRD to confirm that CTO_H presents a single phase (Cu_3_TeO_6_). CTO_H under synchrotron radiation XRD (Figure 1b) shows only characteristic diffraction peaks of Cu_3_TeO_6_ (PDF 01-074-1255). This XRD result indicates that the single-phase CTO is synthesized without incorporation of Na in the crystalline structure of the metal tellurate. In this work, the effect of changing the stoichiometric ratio (2:1 from 3:1) in the synthesis of materials by the hydrothermal method was also studied. The XRD analysis of NTO samples (2:1 and 3:1) (Figure S1a) shows that a change of stoichiometric ratio generated multiphase materials. However, in CTO samples after changing the stoichiometric ratio (2:1 from 3:1), we obtain single-phase Cu_3_TeO_6_, indicating the relevance of the transition metal used. The results obtained by XRD analysis show that the stoichiometric ratio of Ni(NO_3_)_2_·6H_2_O or Cu(NO_3_)_2_·3H_2_O with H_6_O_6_Te is a key factor to obtain single-phase M_3_TeO_6_ (M = Ni or Cu).

To compare the hydrothermal methodology with co-precipitation methodology, the metal tellurates were synthesized by co-precipitation (NTO_CP and CTO_CP) methodology and they were analyzed by lab-source XRD (Figure 1a,b). The NTO_CP sample (Figure 1a) presents mainly the characteristic peaks of the phase Ni_3_TeO_6_. However, in this methodology is also observed a peak at 2θ = 18.46°, indicating that Na has been incorporated in the crystalline structure of Ni_3_TeO_6_ forming the crystal phase Na_2_Ni_2_TeO_6_ (PDF 00-058-0052), as it was observed previously in the literature for materials synthesized by solid-state methodology.^42,43^ This effect is also observed in CTO_CP (see Figure 1b), where the material, prepared by coprecipitation, shows the characteristic peak (2θ = 18.94°) of the crystalline metal tellurate Na_2_Cu_2_TeO_6_^42^ (PDF 04-011-7562). This evidence makes clear that the co-precipitation approach at pH = 7 using NaOH favors the incorporation of Na in the crystal structure of the metal tellurates forming multiphase compounds. Therefore, the hydrothermal synthesis benefits the pure metal tellurates using NaOH as an additive compared to other conventional methods, e.g., co-precipitation synthesis. The pristine metal tellurates synthesized using NaOH by hydrothermal synthesis might be achieved due to the benefits of using hydrothermal synthesis.^44^ The high pressure and temperature generated in the hydrothermal method than the co-precipitation method might change the nucleation, self-assembly, and growth of the crystals, obtaining new and more crystalline phases and different morphologies, as was previously reported in ceramic synthesis.^45,46^ In the hydrothermal methodology performed in this work, the solution with precursors was incorporated in the autoclave after only 5 min of addition of NaOH. Then, the contact with Na^+^ and another cation (Te^6+^ and Ni^2+^) is low before the ions started the nucleation to perform the self-assembly and crystal growth due to the high temperature and pressure inside of the autoclave that favor the nucleation kinetic.^44^ However, the co-precipitation method was performed for 8 h at 80 °C at ambient pressure in stirring; this process helps to hold the required cations close together in the reaction medium, letting the contact and the coordination between the different ions,^47^ including Na^+^. Then, Na^+^ might be incorporated in the structure of the final product more easily than in the hydrothermal method.

To clarify the crystal structures of hydrothermally synthesized NTO and CTO, Rietveld refinements were conducted on the SXRD profiles (see Figure 1c,d). Results show a good fit to a rhombohedral (space group: *R3*) Ni_3_TeO_6_ with lattice constants *a* = *b* = 5.109 Å and *c* = 13.7549 Å and a cubic (space group: *Ia-3*) Cu_3_TeO_6_ with lattice constants *a* = *b* = *c* = 9.5350 Å. Tables S.1 and S.2 summarize the refined unit cell structural parameters and occupation of atoms, respectively.

**Figure 2:**
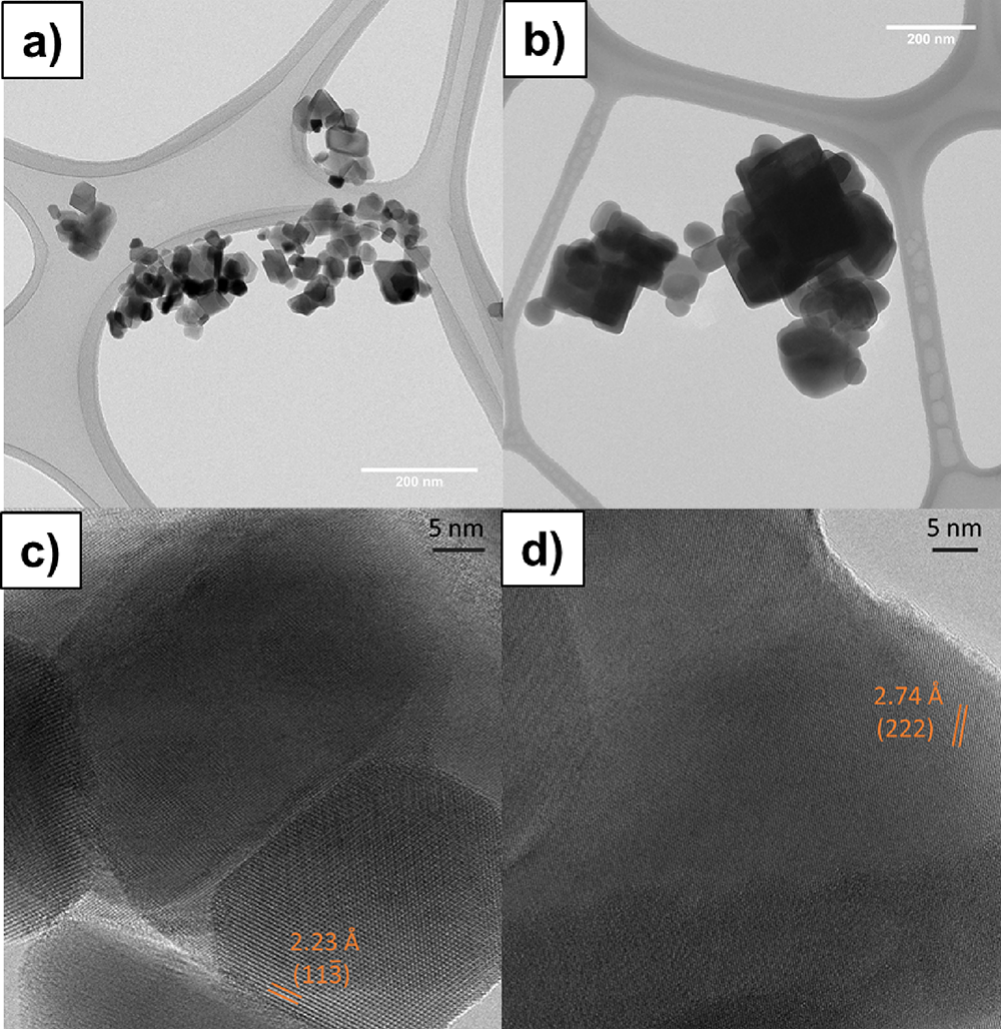
Transmission electron microscopy images for synthesized NTO_H and CTO_H samples; (a) NTO_H samples at low resolution (scale bar: 200 nm), (b) CTO_H samples at low resolution (scale bar: 200 nm), (c) NTO_H samples at high resolution (scale bar: 5 nm), and (d) CTO_H samples at high resolution (scale bar: 5 nm).

The morphology of the metal tellurates prepared in this work by hydrothermal synthesis (NTO_H and CTO_H) was studied by TEM and SEM analyses. TEM analysis (Figure 2a,b) reveals that the metal tellurate materials based on Ni and Cu are present in the form of nanoparticles (NPs) or particles. NTO_H has a heterogeneous morphology based on NPs, as shown in Figure 2a, with an average size of 37 nm (Figure S.2a). The CTO_H sample also presents a varied morphology (Figure 2b), although a cubic NP predominates with an average size of 140 nm (see Figure S.3b). SEM images of NTO_H (Figure S3a) also disclose the presence of NPs. CTO_H presents a heterogeneous morphology as the NTO_H sample; nevertheless, the size of the CTO_H particles (Figure S2b) is bigger than that of the NTO_H sample, confirming the observation obtained by TEM. HR-TEM images (Figure 2c,d) show the high crystallinity of the metal tellurates. NTO_H (Figure 2c) presents a d spacing of ∼2.23 Å corresponding to the (113̅) plane for Ni_3_TO_6_ (PDF 04-009-2820), and a d spacing of ∼2.74 Å is detected for CTO_H (Figure 2d), corresponding to the (222) plane for Cu_3_TeO_6_ (PDF 01-074-1255). The porous texture of the synthesized samples was investigated by N_2_ adsorption measurements (Figure S4). The N_2_ physisorption isotherm of NTO and CTO shows a typical type II isotherm, indicative of a non-porous solid.^48^ The NTO sample presents hysteresis in the high relative pressure range due to interparticle adsorbate condensation. The latter represents a confirmation of the small size of the NTO particles, as observed by TEM analysis.^49^Table S.3 includes the textural properties, which are similar for both materials. Indeed, the two MTOs have a low BET surface area, being non-porous as shown by the N_2_ physisorption isotherm. The NTO_H sample presents a greater value of S_BET_ (12.5 m^2^/g) than CTO_H (7.4 m^2^/g), indicating a higher surface area owing to its lower particle size, evidenced by TEM and SEM analyses (Figure 2 and Figure S.3).

**Figure 3:**
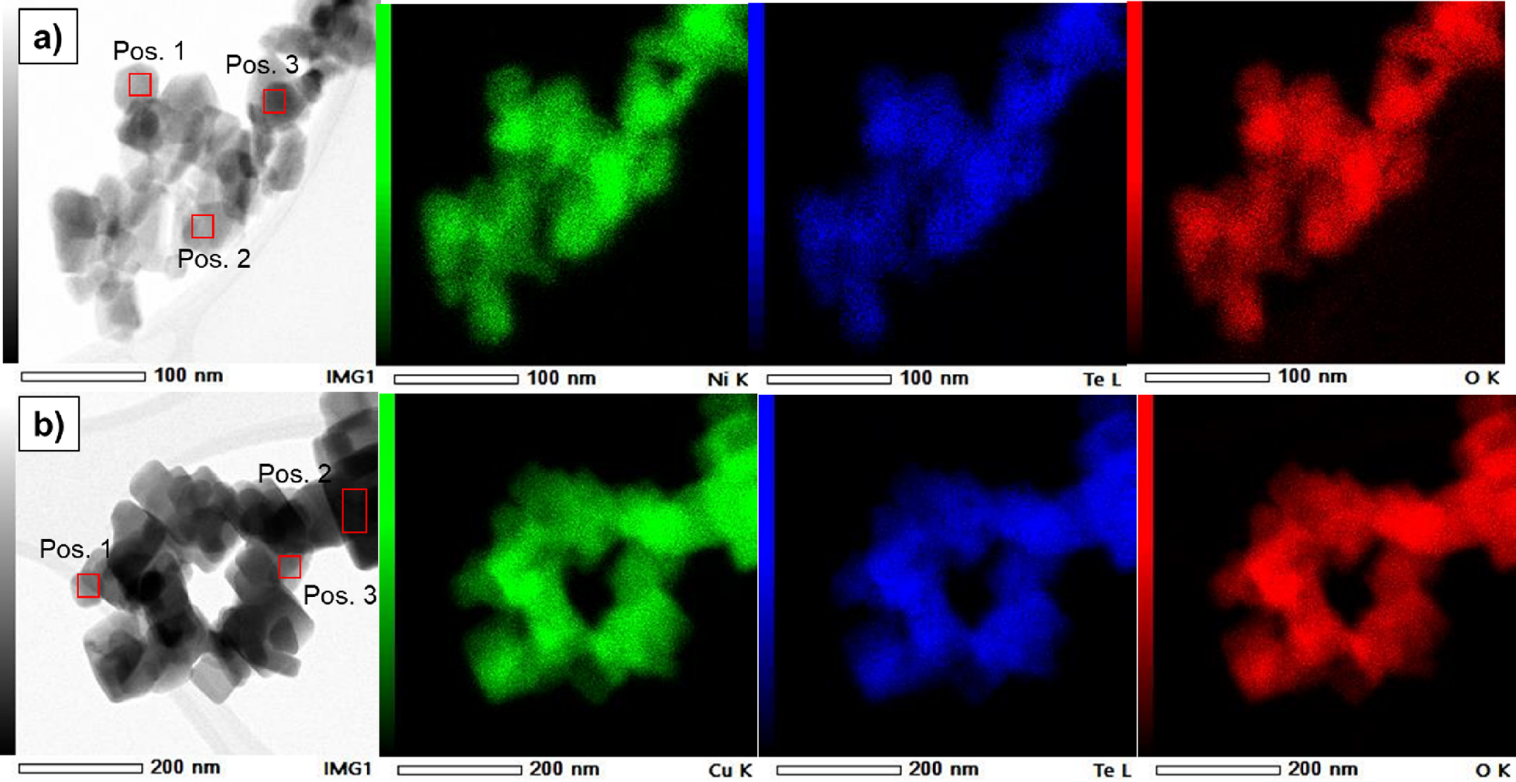
STEM image and corresponding EDS mapping images. (a) STEM images of NTO_H and elemental mapping images of Ni (green), Te (blue), and O (red) elements. (b) STEM images of CTO_H and elemental mapping images of Cu (green), Te (blue), and O (red) elements.

The elemental distribution of the NTO and CTO samples is characterized by using energy-dispersive X-ray spectroscopy (EDS) combined with scanning TEM (STEM-EDS), as depicted in Figure 3. STEM-EDS analysis of the NTO_H (Figure 3a) and CTO_H (Figure 3b) samples shows only presence of Ni or Cu (green), Te (blue), and O (red) elements, indicating that the Na element is not incorporated in the structure of this material, as was observed in the XRD pattern. Moreover, the quantitative analysis by EDS in three different positions (Table S.4) of both samples indicates that the relative amount of the elements M (Ni or Cu), Te, and O is very close to the stochiometric ratio (M_3_TeO_6_).^13,50^ This is another proof that the desired tellurates (M_3_TeO_6_) have been obtained without incorporation of Na in the structure.

**Figure 4:**
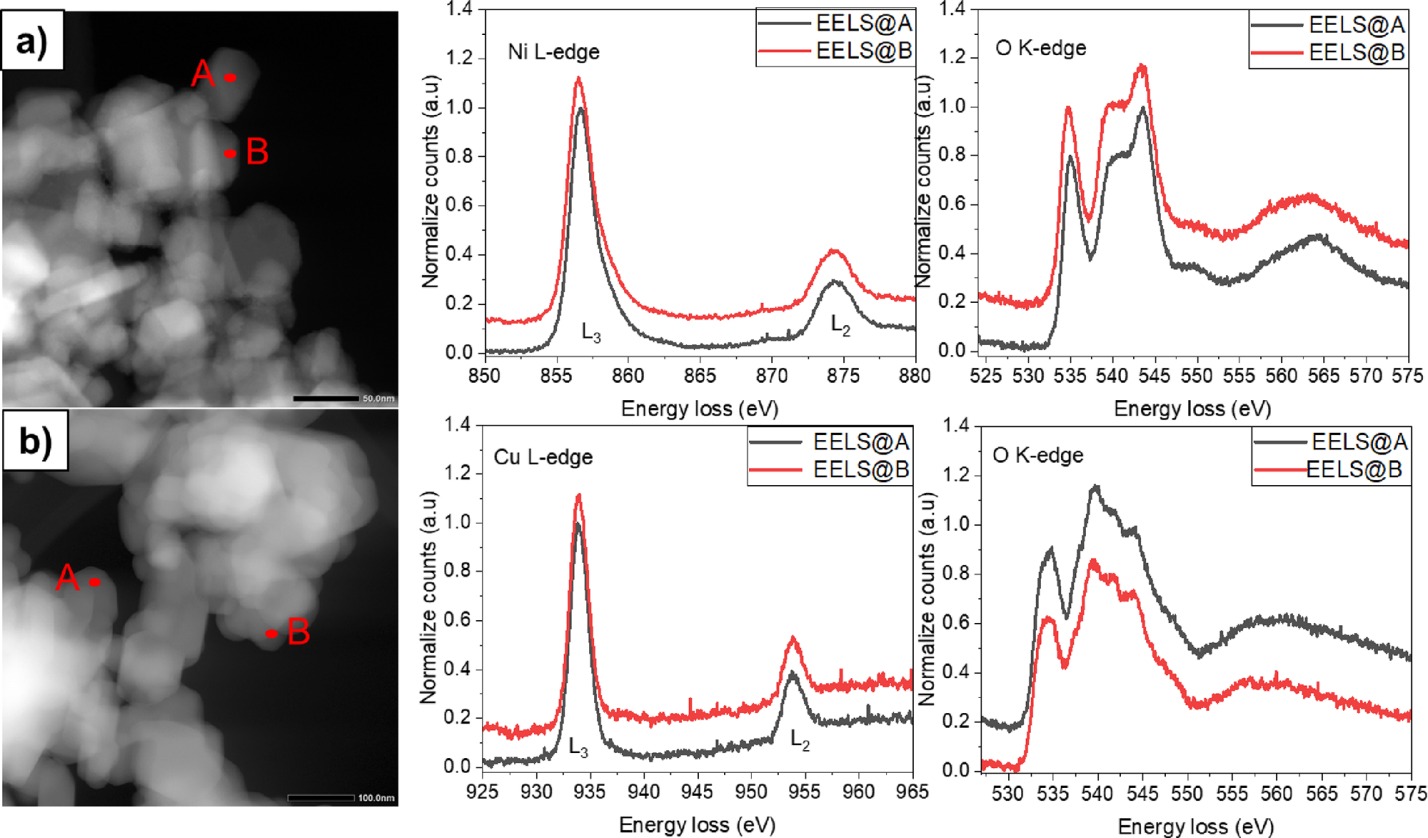
STEM image and corresponding EELS spectra at the marked (A and B in the image) positions for (a) NTO_H and (b) CTO_H.

The EELS spectra of the two selected nano-crystals of NTO (Figure 4a and Figure S.5a) and CTO (Figure 4b and Figure S.5b) correspond to spectroscopic signatures of O K, Te M, and Ni or Cu L edges, indicating the presence of oxygen, Te, nickel, or cooper elements. Moreover, in the NTO sample, a peak is visible in proximities of 856 and 873 eV, which may correspond to the Ni L_2,3_ edge related to the presence of Ni^2+^ in the NTO sample.^39,51^ The oxygen K-edge EELS spectrum of the NTO sample shows a pre-edge feature centered at ∼530 eV, resulting from transitions to unoccupied 3d metal orbitals, which are hybridized with the O 2p orbital.^39,52^ In the oxygen K-edge EELS spectrum is also observed a broader band in the 535–550 eV range. The spectral intensity above the pre-edge is associated with states that have an O 2p-hybridized character with unoccupied 4s and 4p metal orbitals and Te 5pd orbitals.^53,54^ The CTO_H sample shows two peaks centered in 933 and 953 eV corresponding to the Cu L_2,3_ edge due to the presence of Cu^2+^.^55^ As in the NTO sample, the oxygen K-edge EELS spectrum for the CTO sample shows a pre-edge feature centered at ∼530 eV and a broader band in the 535–550 eV range, indicating the same effect. However, the pre-edge feature centered at ∼530 eV and a broader band in 535–550 eV range show some discrepancy with respect to the sample NTO, as a consequence of different bond lengths and geometrical coordination in the two samples. The latter is determined by the presence of different transition metals (Ni or Cu) and crystalline structures, as shown in the XRD pattern and widely discussed in the literature.^7,11^

To confirm the results obtained by EELS analysis, the nanomaterials synthesized by hydrothermal methodology were characterized by XPS (see Figures S6 and S7). The XPS analysis of the 2p level for the NTO_H sample (Figure S.6a) shows a peak at 855.1 eV and a satellite peak at 861.1 eV corresponding to Ni^2+^. The XPS spectra of Ni 2p show another peak at 858.1 eV, which might indicate the presence of Ni(OH)_2_ on the surface of the semiconductor^56^ due to the use of hydrothermal synthesis, as it was previously reported for the synthesis of inorganic materials.^57^ The Te 3d XPS spectra show two different peaks at 576.1 and 577.9 eV, indicating the presence of Te^4+^ and Te^6+^. This result indicates a mixed valence of Te (Te^4+^ and Te^6+^) in NTO materials, as it was recently reported by Numan et al.^58^ The O 1s XPS spectra show a main peak at 530 eV assigned to the oxygen present in Ni_3_TeO_6_ lattices; another shoulder is observed at 531.3 eV, indicating the possible presence of −OH on the surface of NTO_H or oxygen vacancies to compensate the charge valence.^58^ The sample CTO_H presents the divalent Cu^2+^, since the XPS analysis of the Cu 2p level (Figure S.7a) shows a peak at 934.4 eV and a satellite peak at 940 eV.^50^ The CTO_H sample also presents Cu(OH)_2_ because Cu 2p XPS analysis shows another peak at 858.1 eV,^59^ as it happens in NTO_H samples since both samples were synthesized using the same methodology. The analysis of the Te 3d XPS spectra for the CTO_H sample is difficult due to the Cu Auger peak appearing at the same range (569.2 eV). To solve this problem, the Cu Auger peak was fitted to obtain a correct background to successfully perform the analysis of Te 3d XPS peaks. The Te 3d XPS spectra show two different peaks at 576.48 and 577.3 eV, indicating the presence of Te^4+^ and Te^6+^, as it was observed in NTO_H samples. As it was observed in NTO_H, the O 1s XPS spectra of CTO_H show a main peak at 530.46 eV assigned to the oxygen present in Cu_3_TeO_6_ lattices; also, the presence of a shoulder is observed at 531.3 eV indicating the possible presence of −OH on the surface of CTO_H or oxygen vacancies. The results of XPS show that the MTO NPs synthesized in this work revealed that Ni or Cu present +2 valence and Te a mixed valence in nature due to possible oxygen vacancy, as was recently reported.^58^ Moreover, the MTO NPs crystallize in a single phase without incorporation of Na, as per our PXRD and EDS results.

**Figure 5:**
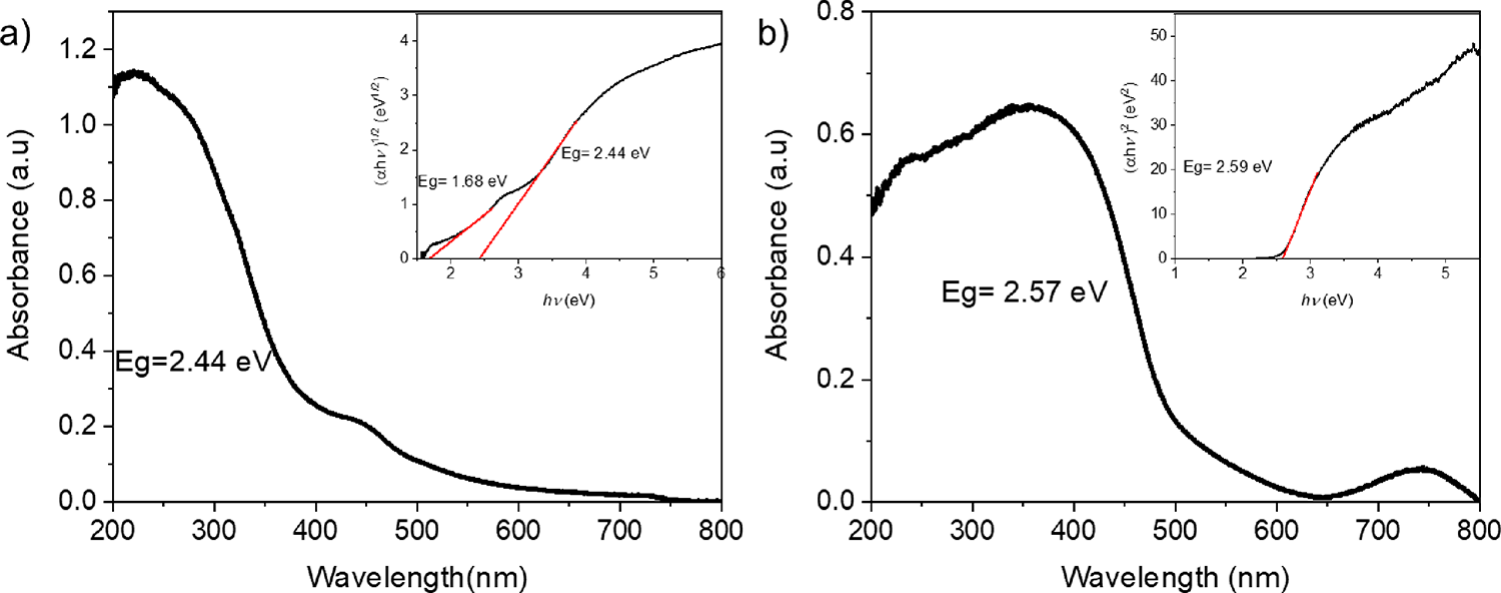
UV–vis spectra of (a) NTO_H and (b) CTO_H. The insets show the bandgap energy (Eg) calculation from the Tauc plot.

The UV–vis spectra of NTO_H and CTO_H are shown in Figure 5. Both metal tellurates display an abrupt cutoff, as common in semiconductors.^50^ The absorption spectrum of NTO_H is in the range of 200–500 nm with two main bands. The second band corresponds to the three-spin allowed d–d transition of the Ni^2+^ ions found in the structure of the material.^13,60^ The CTO_H sample shows two adsorption ranges. The first one is in the range of 200–500 nm due to its semiconductor properties; meanwhile, the second absorption broad band (650–800 nm) corresponds to the d–d transitions from the Cu^2+^ ions presented in the structure of the material.^50^ The bandgap (Eg) of NTO_H and CTO_H was determined by Tauc plot analysis^61^ (Figure 5). The NTO_H and CTO_H samples present a band gap of 2.44 and 2.59 eV, respectively, comparable to the values present in the literature.^13,50^ The Eg calculated for both metal tellurates at the nanoscale indicates that the hydrothermal methodology does not modify the optical properties of these semiconductors. Moreover, this result shows that metal tellurate nanomaterials synthesized by hydrothermal methodology possess promising optical properties and absorption in the visible range for their use in different applications.

### Magnetic Properties

**Figure 6:**
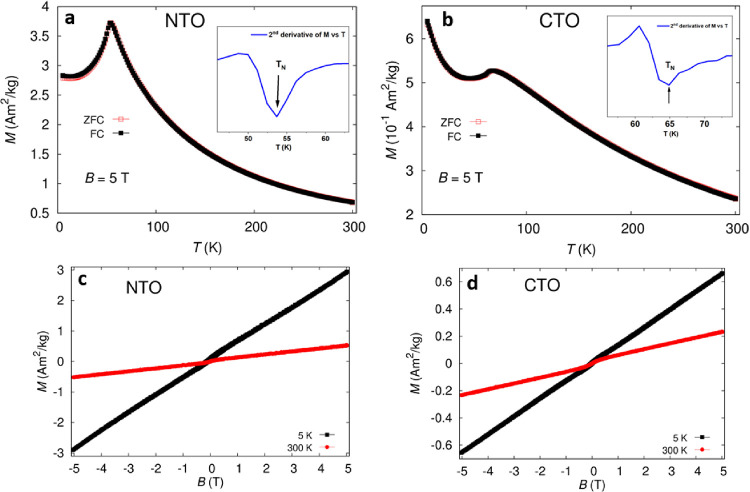
Magnetic domain evolution behavior studies under external field stimuli of temperature and magnetic field. (a, b) Temperature-dependent magnetization for (a) NTO and (b) CTO. (c, d) Field-dependent magnetization at 5 and 300 K for (c) NTO_H and (d) CTO_H.

Figure 6a,b shows the magnetization vs temperature characteristics of NTO_H (*T*_N_ ∼ 54 K) and CTO_H (*T*_N_ ∼ 64 K), respectively, which are in line with the previous reports for powder as well as single-crystal enhancement of AFM interaction and transition temperature in M_3_TeO_6_ systems (see Table S.5).^2,4,62^ The results indicate that both metal tellurates are paramagnetic (PM) in nature at room temperature. Temperature-dependent magnetization *M*(*T*) measured under the field-cool (FC) and zero-field-cool (ZFC) protocol for both metal tellurates show PM behavior (Figure 6a,b). For NTO_H, the AFM coupling starts below *T*_N_ of ∼57 K (see Figure 6a). Similarly in CTO_H, magnetization increases with decreasing temperature down to 70 K and again below 25 K. The MT curve shows a drop in *M* at around 68 K, indicating a clear AFM coupling below this temperature. However, below 26 K, the PM phase clearly dominates, decreasing the AFM behavior at the same time (see Figure 6b). After comparing with the magnetization of single-crystal data of these two compounds, magnetization curves indicate the similar behavior of NTO_H and CTO_H with reported single crystals, showing the same intrinsic characteristics. Moreover, our NTO_H and CTO_H exhibit slightly higher AFM transition temperatures compared to those in literatures. Magnetic field dependencies at both 5 and 300 K for NTO_H and CTO_H show an increase in their magnetization with increasing external magnetic field (Figure 6c,d), being in line with the results shown in the literature.

Also, the magnetic measurement shows the ZFC and FC curves at a low magnetic field of 10 mT (Figure S.8) showing deviation from each other, due the spin-glass or cluster-glass state within the PM state. This effect is described previously by Panneer Muthuselvam et al. for nanograin NTO, where the bulk and shell make different contributions to the superposition of the magnetic moment, as can be seen in these kinds of measurements.^62^ In FC curves measured at a relatively small field, AFM coupling is relatively strong in ZFC curves, but upon FC, a clear increase in *M* can be seen below 57 K. Both metal tellurates present a mechanism related to the nanograined system,^62^ due to the NP size of these materials, which are 37 and 140 nm for NTO_H and CTO_H, respectively (see Figure 2a,b). However, the main different behavior in magnetic properties of both metal tellurates is related in that the CTO_H sample does not have any atoms with unfilled shells, whereas NTO_H has Ni.

### Photoconductivity of Metal Tellurates for Their Potential Use in Photodetection

In general, nonmetal elements with a relatively high refractive index should be photoconductive materials.^63,64^ This concept has already been confirmed in terms of photoconductive effect in Te films,^65^ Te nanowires,^66^ and CdTe nanowires.^67^ Recently, CsCdInQ_3_ (Q = Se, Te) has also been reported to be a photoconductive compound. Although a few reports are available concerning the photo response of Te-based alloy and composition, a similar observation is missing for any of the MTO combination. To guess the photoconductive effect in MTO, we first calculated the refractive index of NTO and CTO from their measured band gaps and several established relations (see Figure S.9) and found a qualitative lead toward their possible photo response characteristics. To confirm the same, we measured the photo response of these materials.

**Figure 7:**
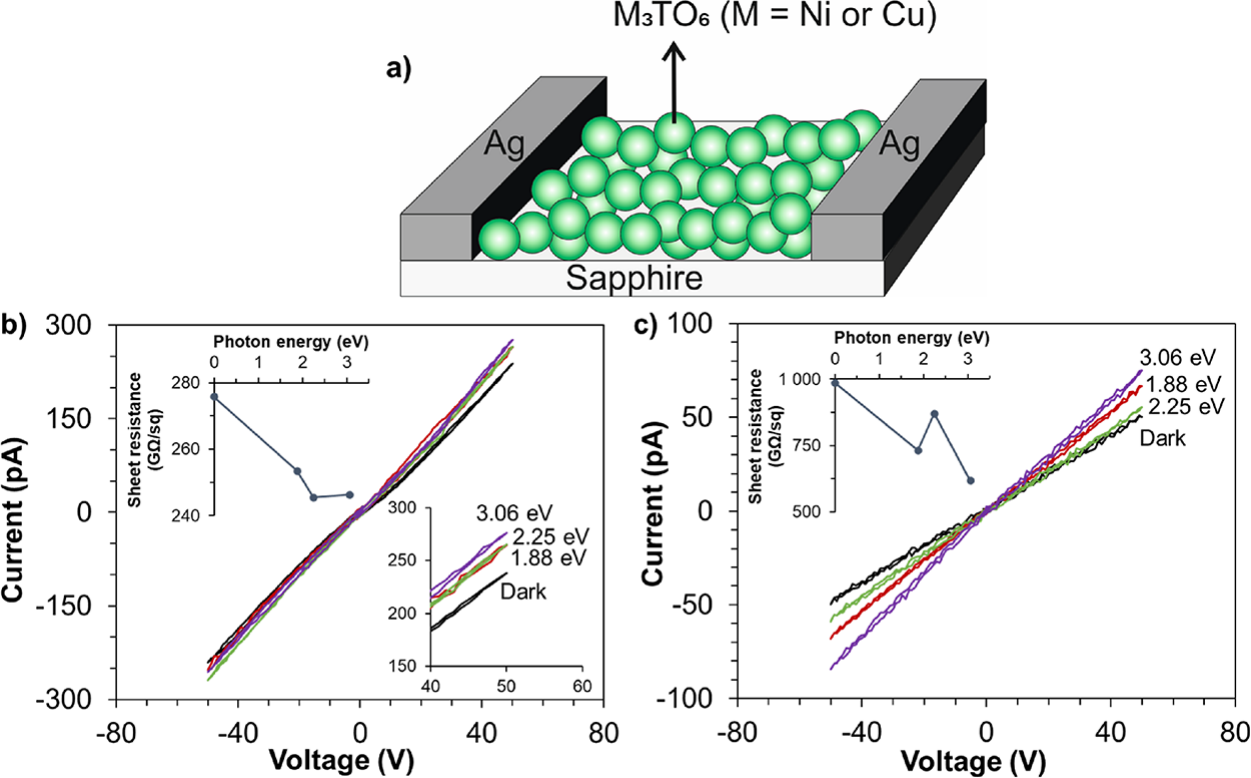
Photoelectrical performance of the nano-MTO-based devices. (a) Schematic of the device structure, and *I*–*V* curves of (b) NTO and (c) CTO obtained under different illumination conditions. The insets of (b) and (c) show the dependence of calculated sheet resistance on incident photon energy (0 eV represents the dark).

Figure 7a illustrates the device configuration used for the *I*–*V* curve measurement, which also serves as a proof-of-concept demonstration of a potential photodetection device. Figure 7b,c shows the *I*–*V* curves measured on the NTO and CTO devices, respectively. Both samples exhibited photoconductivity where the sheet resistance of the NTO decreased by 14% compared with the values obtained in the dark and when exposed to violet light (3.06 eV). The response of CTO was even more obvious with a nearly 40% decrease of sheet resistance under violet light. The behavior of photoconductivity was in line with the UV–vis spectra (Figure 5) and thus confirms both NTO and CTO to be proper semiconductors with moderate band gaps. The origin of the photoconductivity observed in the NTO and CTO samples (Figure 7a,b) is due to the illumination of these semiconductors with an energy higher than the band gap (2.44 eV for NTO and 2.59 eV for CTO), which generated electron–hole pairs. This resulted in increased electrical conductivity and is the foundation of the photodetection function. Alongside the previously reported photodetection capabilities of perovskites and metal oxide semiconductors,^68−70^ the test performed in this work showed that the MTO devices were able to work effectively between the green and violet light (photon energies of 2.2–3.1 eV) thanks to their moderate band gaps. This opens doors to a future violet photodetector made from the MTO nanostructures.

## Conclusions

In conclusion, two metal tellurate M_3_TeO_6_ (M = Ni (NTO) or Cu (CTO)) at the nanoscale have been successfully synthesized via hydrothermal synthesis using NaOH as the additive. A promising hydrothermal methodology was presented, which allows the synthesis of MTOs without the incorporation of Na in the structure, resulting in the formation of Na_2_M_2_TeO_6_ (NaMTO). On the contrary, the co-precipitation synthesis led to the undesired synthesis of NaMTO, as demonstrated by XRD and EDS characterization. The metal tellurates (NTO and CTO) obtained by this methodology are composed by NPs with an average size of 37 and 140 nm, respectively. NTO and CTO samples prepared in this work with NP morphology are semiconductors with band gaps of 2.44 and 2.56 eV, and both exhibited photoconductivity. In addition, NTO and CTO prepared in this work using hydrothermal synthesis present magnetic properties, with *T*_N_ of 57 and 68 K, respectively. The comparable magnetic properties and the semiconductor properties with photoconductivity of the metal tellurates synthesized by hydrothermal methodology using NaOH to the same metal tellurates prepared by a conventional approach, e.g., solid state, evidence that the use of hydrothermal methodology is an innovative methodology for the synthesis of this class of nanomaterials. This scenario opens the possibility of synthesizing new materials within this metal tellurate family using hydrothermal synthesis with controlled coordination, homogeneous size crystal distribution, and different morphologies for their use in different applications. Indeed, this work serves as a preliminary proof of concept for potential application of MTO nanomaterials as photodetectors due the photoconductivity results obtained.

## Supporting Information Available

Rietveld refinement and atomic occupancy results of SXRD data, size distribution histogram, SEM images, N_2_ isotherms at 77 K and textural properties, EDS calculation of STEM-EDS images, EELS spectra for the Te M edge, and temperature-dependent magnetization (PDF)
