# Supplementary material for: Hydrothermal Synthesis of Ni3TeO6 and Cu3TeO6 Nanostructures for Magnetic and Photoconductivity Applications
Source: ACS Appl Nano Mater. 2023 Mar 9;6(6):4887–97. doi: 10.1021/acsanm.3c00630 (PMC10043876; doi:10.1021/acsanm.3c00630)
Supplement: Supplementary file 1 — an3c00630_si_001.pdf [file an3c00630_si_001.pdf]

Supporting Information

Hydrothermal Synthesis of Ni_3TeO_6 and Cu_3TeO_6 Nanostructures for Magnetic and Photoconductivity Applications

Javier Fernández-Catalá[1,2], Harishchandra Singh[1]*, Shubo Wang[1], Hannu Huhtinen[3], Petriina Paturi[3], Yang Bai[4], Wei Cao[1]*

[1]Nano and Molecular Systems Research Unit, University of Oulu, FIN-90014, Finland.

[2] Materials Institute and Inorganic Chemistry Department, University of Alicante, Ap. 99, E-03080 Alicante, Spain.

[3]Wihuri Physical Laboratory, Department of Physics and Astronomy, FIN-20014 University of Turku, Finland.

[4]Microelectronics Research Unit, Faculty of Information Technology and Electrical Engineering, University of Oulu, FI-90570 Oulu, Finland.

Corresponding authors: Harishchandra Singh; email: harishchandra.singh@oulu.fi and Javier Fernández-Catalá; email: j.fernandezcatala@ua.es

Table of Contents

Table S.1. Rietveld refinement results of SXRD Data for MTOs. S-3

Table S.2. Atomic occupancy obtained of the SXRD data for MTOs. S-3

Figure S.1. PXRD patterns for synthesized NTO and CTO samples using different stoichiometric ratios of Ni and Te precursors using hydrothermal synthesis: (a) NTO samples, and (b) CTO samples. S-3

Figure S.2. Histograms by counting 100 particles of (a) NTO_H and (b) CTO_H. S-4

Figure S.3. SEM images of (a) NTO_H and (b) CTO_H. S-4

Figure S4. N_2 isotherms at 77 K for the samples prepared in this study. S-5

Table S.3. Textural Properties of the NTO_H and CTO_H. S-5

Table S.4. EDS spectroscopy corresponding of STEM-EDS images for NTO and CTO. S-6

Figure S.5. EELS spectra at two positions marked in STEM Figure 3 (a,b), corresponding (a) NTO_H and (b) CTO_H for Te M-edge. S-6

Figure S.6. XPS spectra of a) Ni 2P, b) Te 3d and O 1s from NTO_H sample. S-7

Figure S.7. XPS spectra of a) Cu 2P, b) Te 3d and O 1s from CTO_H sample. S-7

Table S.5. Comparison of antiferromagnetic (AFM) transition temperature over Ni_3TeO_6 and Cu_3TeO_6 materials. S-8

Figure S.8. Temperature-dependent magnetization for: a,) NTO_H, and b) CTO_H for low magnetic field. S-8

Figure S.9. Refractive index of investigated NTO and CTO. S-9

Table S.1. Rietveld refinement results of SXRD Data for MTOs.

	**Lattice constant (Å)**	**V (Å^3)**	**Avg. M-O**	**Te-O (Å)**	**R_{wp} (%)**	**R_p (%)**
**NTO**	*a=5.1009, c=13.7549*	309.94	2.02	1.96	8.81	6.61
**CTO**	9.5350 (*a=b=c)*	866.88	2.10	1.95	7.79	4.24

Table S.2. Atomic occupancy obtained from Rietveld refinement of the SXRD data for MTOs.

	Atom	Site	x	y	z	Occu.	B (Å^2)
NTO	Ni1	3a	0.0000	0.0000	0.3566	1.000	0.321
	Ni2	3a	0.0000	0.0000	0.6486	0.986	0.339
	Ni3	3a	0.0000	0.0000	0.8511	1.000	0.121
	Te	3a	0.0000	0.0000	0.1554	1.000	0.205
	O1	9b	0.2890	0.0000	0.2465	1.000	0.240
	O2	9b	0.67951	0.0000	0.7512	1.000	0.750
CTO	Cu	24d	0.9685	0.1732	0.3972	0.958	0.395
	Te	48e	0.2500	0.2500	0.2500	0.929	0.229
	O	8b	0.3681	0.1732	0.3972	1.000	0.553

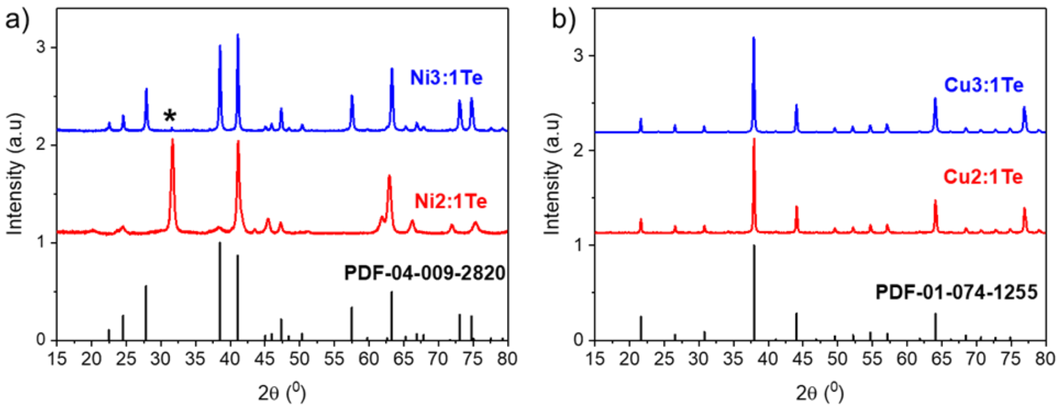

Figure S.1. PXRD patterns for synthesized NTO and CTO samples using different stoichiometric ratios of Ni and Te precursors using hydrothermal synthesis: a) NTO samples, and b) CTO samples.

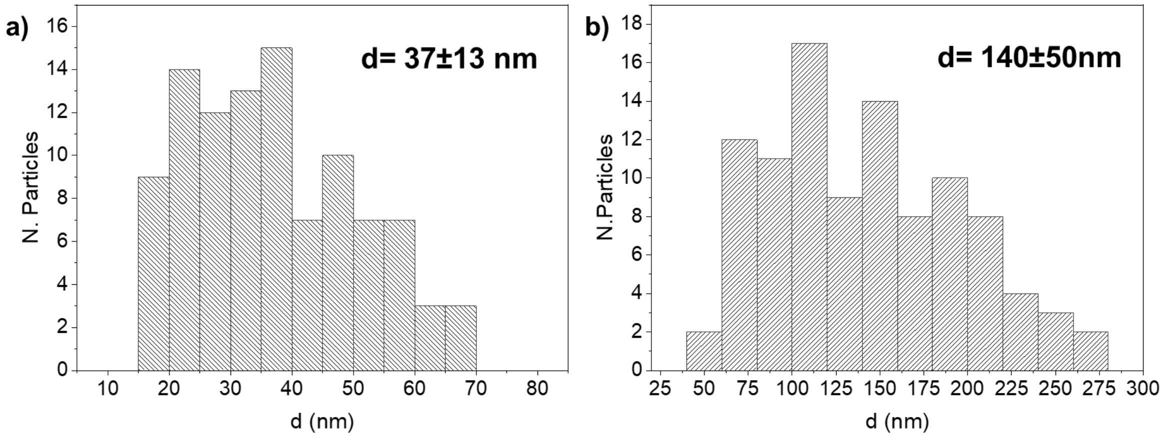

Figure S.2. Histograms by counting 100 particles of a) NTO_H and b) CTO_H.

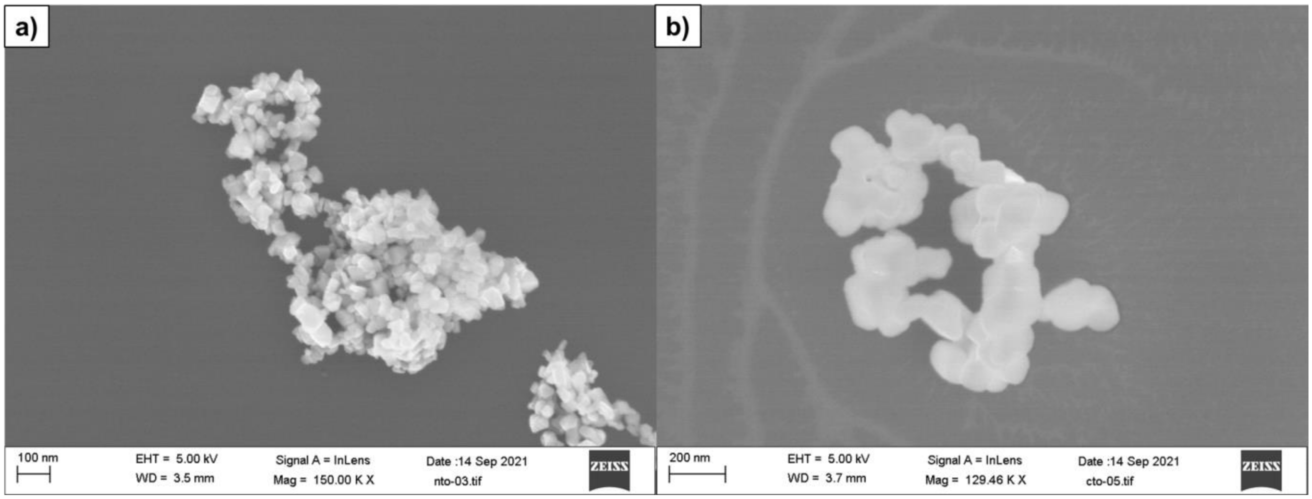

Figure S.3. SEM images of a) NTO_H and b) CTO_H

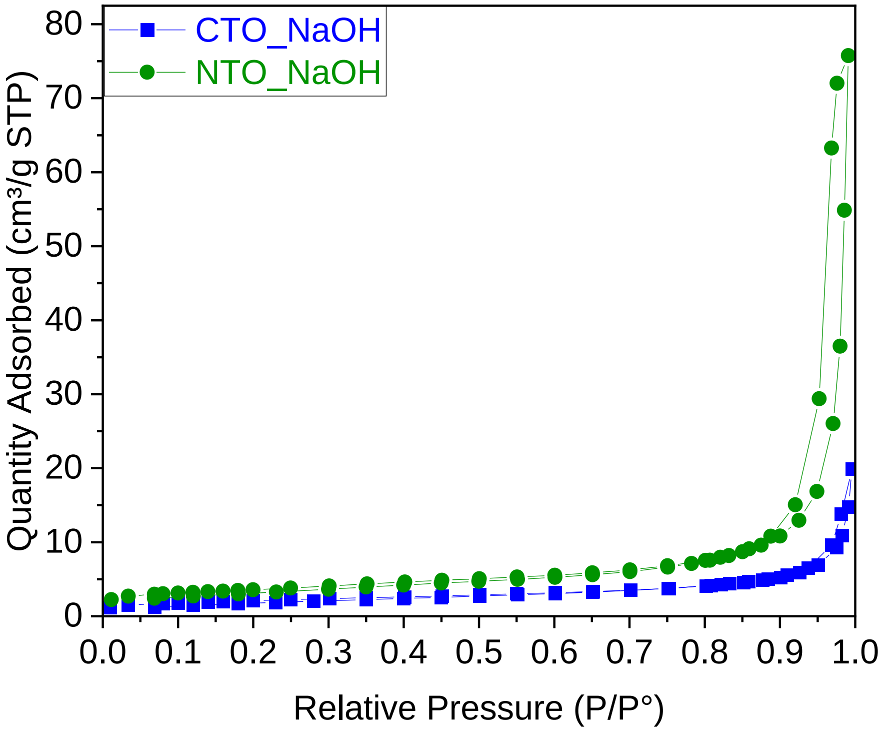

Figure S4. N_2 isotherms at 77 K for the samples prepared in this study: NTO_H, and CTO_H.

Table S.3. Textural Properties of the NTO_H and CTO_H.

Samples	S_{BET} (m^2/g)	$V_{total,0.95}$ (cm^3/g)	V_{N2DR} (cm^3/g)
NTO_H	12.5	0.026	0.0062
CTO_H	7.4	0.011	0.0038

Table S.4. EDS spectroscopy corresponding of STEM-EDS images for NTO and CTO.

Samples	M: Ni/Cu (Atomic %)	Te (Atomic %)	O (Atomic %)
NTO_H Pos.1	27.01	9.79	63.20
NTO_H Pos.2	29.63	9.21	61.17
NTO_H Pos.3	28.06	10.40	61.54
CTO_H Pos 1	31.90	12.22	55.89
CTO_H Pos 2	28.09	10.58	61.33
CTO_H Pos 3	28.89	10.83	60.28

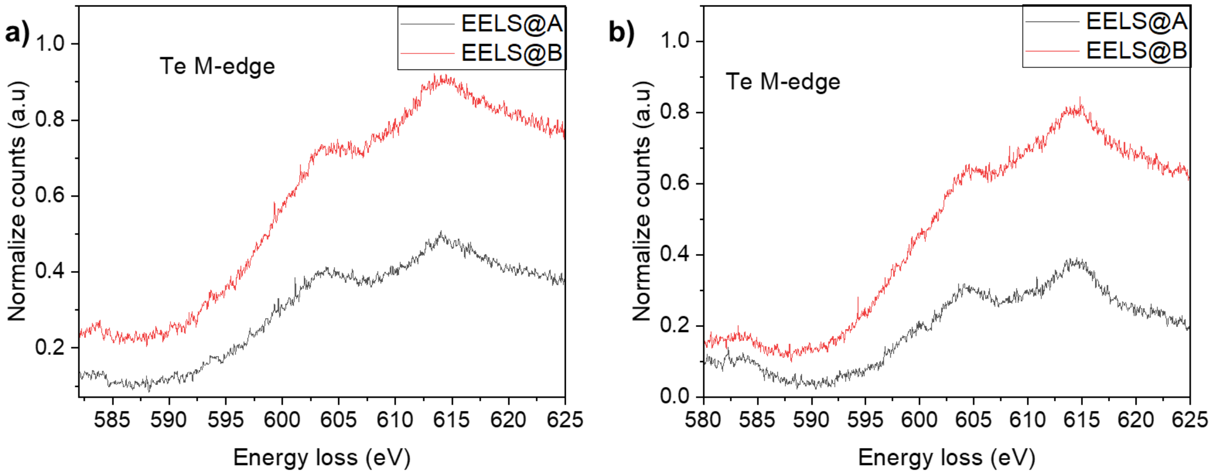

Figure S.5. EELS spectra at two positions marked in STEM Figure 3 (a,b), corresponding a) NTO_H and b) CTO_H for Te M-edge.

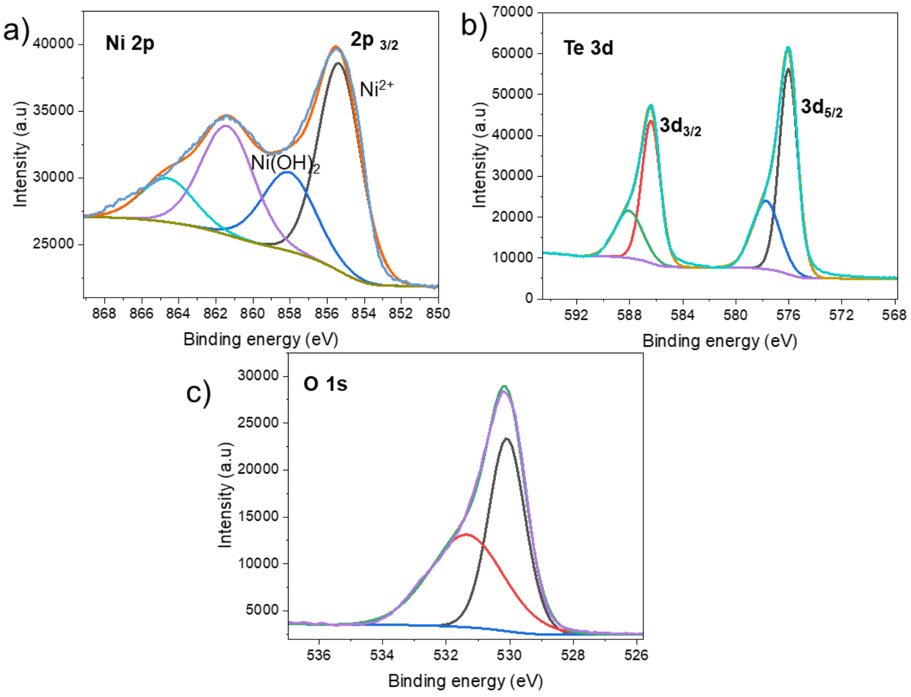

Figure S.6. XPS spectra of a) Ni 2P, b) Te 3d and c) O 1s from NTO_H sample.

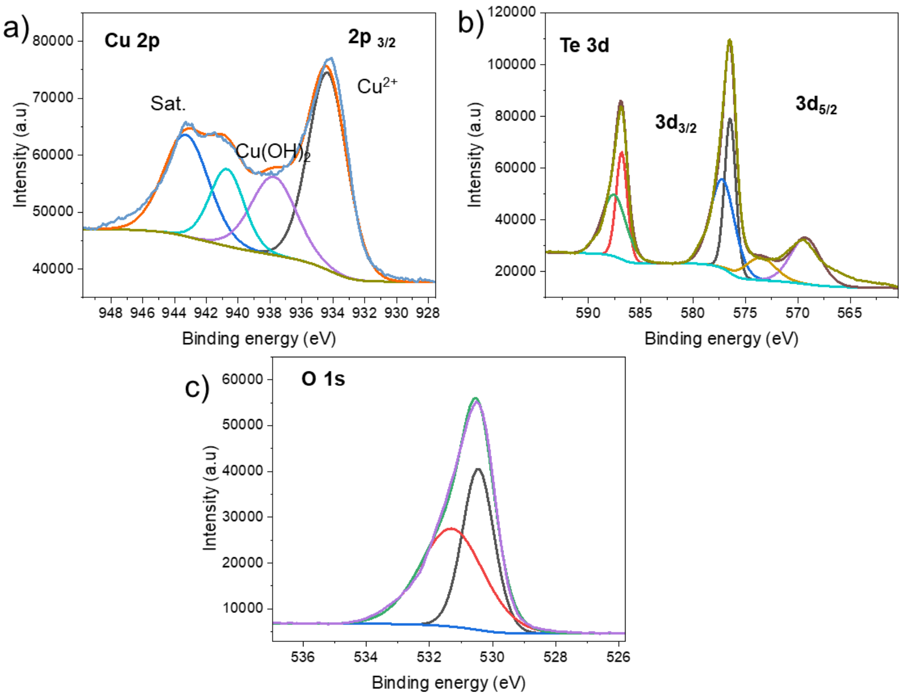

Figure S.7. XPS spectra of a) Cu 2P, b) Te 3d and c) O 1s from CTO_H sample.

Table S.5. Comparison of antiferromagnetic (AFM) transition temperature over Ni_3TeO_6 and Cu_3TeO_6 materials.

Samples	Synthesis method	T_N (K)	References
NTO_H	Hydrothermal	52	This work
Ni_3TeO_6 single crystals	Vapour transport method	52	1
Ni_3TeO_6 single crystals	Solid state reaction	52	2
Ni_3TeO_6 single crystals	Vapour transport method	55	3
Nano-Grain Sized Ni_3TeO_6	Solid state reaction	52	4
CTO_H	Hydrothermal	63	This work
Cu_3TeO_6 single crystals	Solid state reactions	61	2
Cu_3TeO_6 single crystals	Chemical transport method	61	5
Cu_3TeO_6 single crystals	Chemical transport method	63	6
Cu_3TeO6 single crystals	Chemical transport method	61.7	7

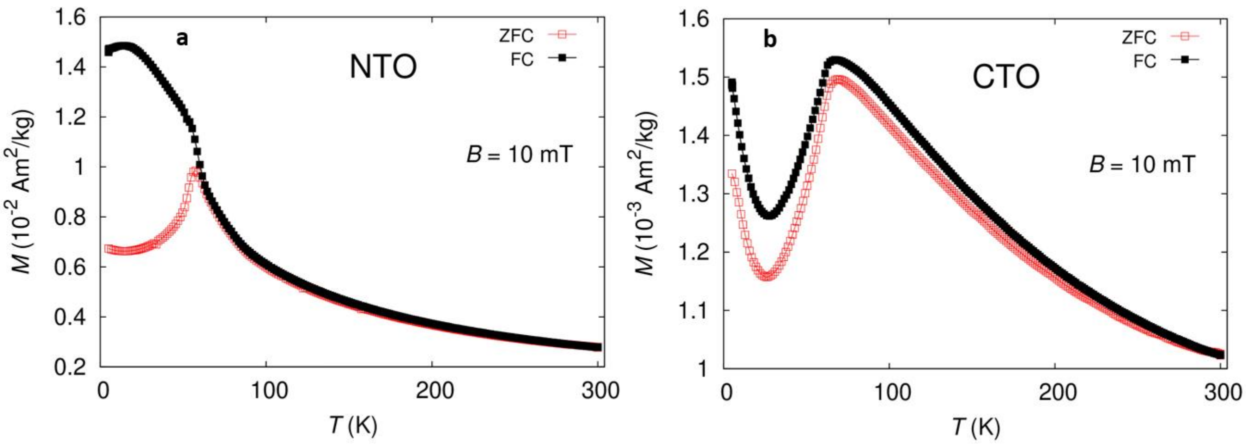

Figure S.8. Temperature-dependent magnetization for: a) NTO_H, and b) CTO_H for low magnetic field.

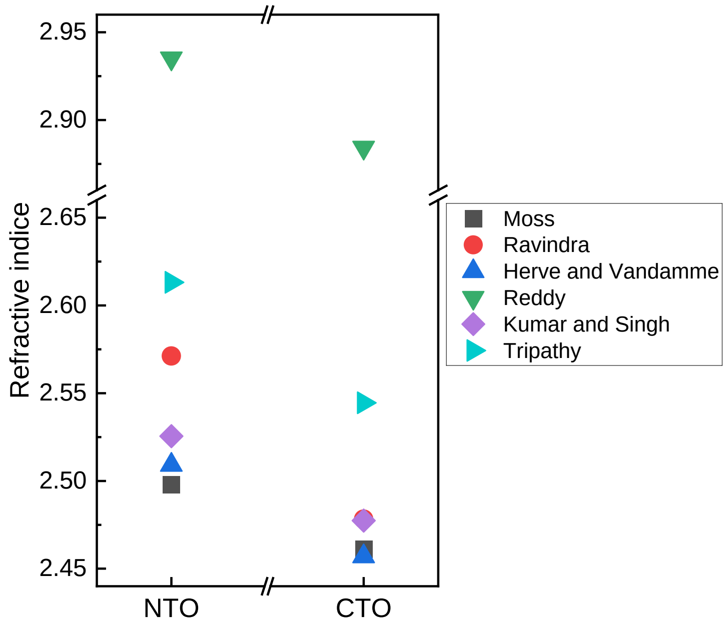

Figure S.9. Refractive index of investigated NTO and CTO (experimentally Eg = 2.44 eV and 2.59 eV, respectively) from using six established relations in the work of Tripathy.[8].

REFERENCES

(1) Kim, J. W.; Artyukhin, S.; Mun, E. D.; Jaime, M.; Harrison, N.; Hansen, A.; Yang, J. J.; Oh, Y. S.; Vanderbilt, D.; Zapf, V. S.; Cheong, S. W. Successive Magnetic-Field-Induced Transitions and Colossal Magnetoelectric Effect in Ni_3TeO_6. *Phys. Rev. Lett.* **2015**, *115* (3), 1–5. https://doi.org/10.1103/PhysRevLett.115.137201.

(2) Mathieu, R.; Ivanov, S. A.; Nordblad, P.; Weil, M. Enhancement of Antiferromagnetic Interaction and Transition Temperature in M_3TeO_6 Systems (M = Mn, Co, Ni, Cu). *Eur. Phys. J. B* 2**013**, *86* (8), 3–6. https://doi.org/10.1140/epjb/e2013-40152-x.

(3) Oh, Y. S.; Artyukhin, S.; Yang, J. J.; Zapf, V.; Kim, J. W.; Vanderbilt, D.; Cheong, S. W. Non-Hysteretic Colossal Magnetoelectricity in a Collinear Antiferromagnet. *Nat. Commun.* **2014**, *5*, 1–7. https://doi.org/10.1038/ncomms4201.

(4) Panneer Muthuselvam, I.; Saranya, K.; Sankar, R.; Bhowmik, R. N.; Kavitha, L.

Experimental Study of Multiple Magnetic Transitions in Micrometer and Nano-Grain Sized Ni_3TeO_6-Type Oxide. *J. Appl. Phys.* **2020**, *128* (12). https://doi.org/10.1063/5.0020807.

(5) Herak, M.; Berger, H.; Prester, M.; Miljak, M.; Živković, I.; Milat, O.; Drobac, D.; Popović, S.; Zaharko, O. Novel Spin Lattice in Cu_3TeO_6: An Antiferromagnetic Order and Domain Dynamics. *J. Phys. Condens. Matter* **2005**, *17* (48), 7667–7679. https://doi.org/10.1088/0953-8984/17/48/017.

(6) Caimi, G.; Degiorgi, L.; Berger, H.; Forró, L. Optical Evidence for a Magnetically Driven Structural Transition in the Spin Web Cu_3TeO_6. *Europhys. Lett.* **2006**, *75* (3), 496–502. https://doi.org/10.1209/epl/i2005-10603-3.

(7) Mansson, M.; Prsa, K.; Sugiyama, J.; Andreica, D.; Luetkens, H.; Berger, H. Magnetic Order and Transitions in the Spin-web Compound Cu_3TeO_6. *Phys. Procedia.* **2012**, *30*, 142-145.

(8) Tripathy, S. K. Refractive Indices of Semiconductors from Energy Gaps. *Opt. Mat.* **2015**, *46*, 240-246. Doi: 10.1016/j.optmat.2015.04.026
